# Synthesis, evaluation of drug delivery potential, and the quantum chemical investigation on a molecular imprinted polymer for quetiapine antipsychotic; a joint experimental and density functional theory study

**DOI:** 10.3389/fchem.2022.1001685

**Published:** 2022-10-14

**Authors:** Seyyedeh Fatemeh Hoseini chehreghani, Parviz Aberoomand Azar, Maryam Shekarchi, Bahram Daraei

**Affiliations:** ^1^ Department of Chemistry, Science and Research Branch, Islamic Azad University, Tehran, Iran; ^2^ Food and Drug Laboratory Research Center, Food and Drug Organization, MOH and ME, Tehran, Iran; ^3^ Department of Toxicology and Pharmacology, School of Pharmacy, Shahid Beheshti University of Medical Sciences, Tehran, Iran

**Keywords:** quetiapine, molecular imprinted polymers, MIP, NIP, DFT, controlled release

## Abstract

In this project, the quetiapine drug was used as the template for synthesis of a molecular imprinted polymer (MIP). The polymerization approach for preparation of this composite was precipitation, where methacrylic acid (MAA), ethylene glycol dimethacrylate (EGDMA), and 2,2-azobisissobutyronitrile (AIBN) were used as the functional monomer, the cross-linker, and the initiator, respectively. Scanning electron microscopy (SEM) showed that the diameter of the nanoparticles is about 70 nm. The adsorption rates of quetiapine to the MIP host were evaluated at different pHs, and the results showed that the highest adsorption values were obtained at pH = 7. Moreover, the kinetics of the adsorption process was detected to follow the Langmuir isotherm (R^2^ = 0.9926) and the pseudo-second-order kinetics (R^2^ = 0.9937). The results confirmed the high capability of the synthesized MIPs as pharmaceutical carriers for quetiapine. Furthermore, the kinetics of the drug release from the MIP follows the Higuchi model at the pHs of 5.8–6.8 and the Korsmeyer–Peppas model at the pHs of 1.2–5. Finally, in light of the density functional theory (DFT)-based quantum chemical descriptors, the polymer–quetiapine drug complex was designed and investigated. The results showed that there is a strong interaction between the host (polymer) and the guest (drug) due to several hydrogen bonds and other intermolecular (polar) interactions.

## Graphical Abstract

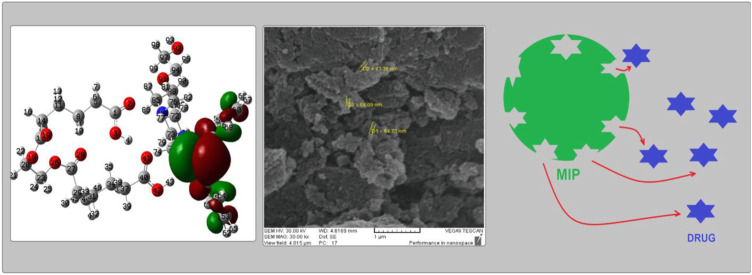

## 1 Introduction

Quetiapine (which has a six-membered heterocyclic ring as a biologically active part of this molecule) is one of the most important atypical antipsychotic drugs which have been used to treat some mental illnesses such as schizophrenia, bipolar disorder, and depression (Poyurovsky et al., 2020). This compound operates to restore the balance of some special chemical neurotransmitters or messengers in the central nervous system (CNS), which improves mood, behavior, and thinking (Garakani et al., 2020). It also works on blocking the receptors of serotonin and dopamine, two important neurotransmitters (Jain, 2019). Due to the wide usage of this compound, some formulations such as standard and slow-release tablets or oral suspensions were prepared commercially (Papazisis and Siafis, 2019). The release rate of the drug compounds into the blood serum, which could be optimized *via* different approaches, has always been an important issue for formulators. It would be more important when the release rate and the bioavailability of a medicinal compound are at a high level, while the half-life and the required dosage of that drug for the body are lower than the standards (Wang et al., 2018). In this regard, a number of methods have been designed for regulation of the release rate of drugs such as the slow-release tablets (Eriksson et al., 2020), osmotic pumps (Shah and Makwana, 2021), molecular imprinted polymers (MIPs) (Motaharian et al., 2020; He et al., 2021), liquid crystals (Bodratti and Alexandridis, 2018), and sol–gels (Lee et al., 2019).

**FIGURE 1:**
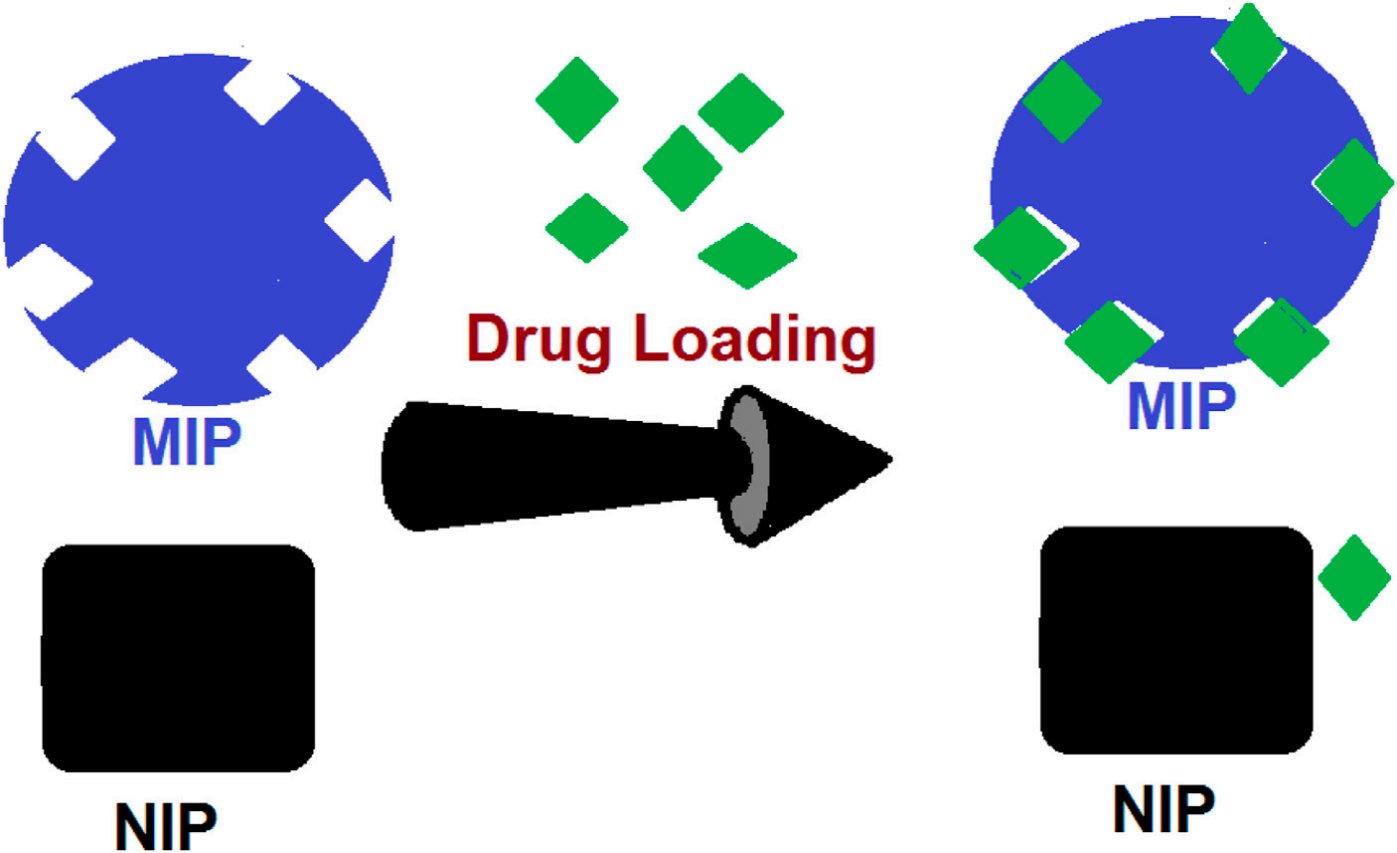
Process of absorption of the drugs by MIP substrates compared to the adsorption by NIP.

Among several invented drug delivery systems, MIPs, these special synthetic polymers which are similar to antibodies (having certain structural space for identifying target molecules), are of the best achievements of the molecular imprinting technology (MIT). These special structures have recently been developed as suitable candidates for drug delivery usage (Zaidi, 2020). In such models, polymers are formed in the presence of a drug as a molecular template (Figure 1) to mimic the real drug receptors. After the polymer is formed, the product is washed several times to prepare the MIP substrates, free of those tinny templates. Hence, the whole structure of the matrix is full of numerous free substrates which would accept guests for further drug delivery (DD) applications (Marcelo et al., 2018). Moreover, such precious nano-sized receptors could be used as a stationary phase for chromatography column packing (Ali et al., 2022), nano-sorbents (Sadegh et al., 2021), actuators (Weber et al., 2018), biosensors (Park et al., 2022), and nano-scale drug delivery machines (Nahhas and Webster, 2021).

In recent decades, MIP materials have been reported to be applied as a new drug delivery system (DDS). Using this technology, medics could create accurate dosages and optimize the release rate of the drugs (Liu et al., 2019).

Thus, in this research, we have used quetiapine as the molecular template to design some MIP granules for further application in the drug delivery system. Scanning electron microscopy (SEM) was used for morphology studies and cross-section of MIPs which indicated that the diameters of the MIPs were about 70 nm. Also, the drug release properties of the composites and the abilities of those for *in vitro* release were investigated by the high-performance liquid chromatography (HPLC) system (Chamberlain et al., 2022; Dadras et al., 2022). Indeed, one of the aims of this research is to investigate the drug adsorption/release behavior using types of kinetic models and to determine its mechanism. One of the other objectives of this research is to understand the nature of this adsorption process in terms of the atom and molecules. There are several examples of the successful use of theoretical calculations for investigation of adsorption processes (Siadati et al., 2016a; Vessally et al., 2017; Rad and Ayub, 2018). Therefore, here, the adsorption process of quetiapine, both by NIP and MIP, was studied by means of the density functional theory (DFT) quantum chemical approach (which has been shown to be trustable and widely applied for studying adsorptions on nano-particles) (Pakravan and Siadati, 2017;‏ Siadati and Mirabi, 2015; Siadati et al., 2016b; Sun et al., 2021). The results of the theoretical calculations support the experimental outcomes, showing that the polymer matrix (the host) could have stronger interactions with quetiapine (the guest).

## 2 Experimental

### 2.1 Materials and reagents

All of the chemical reagents and the required solvents were of analytical grade. Ultra-pure water was obtained by a Milli-Q purification system (Millipore, Bedford, MA, United States). The chemical compounds, materials, and reagents containing ethylene glycol dimethacrylate (EGDMA), methacrylic acid (MAA), 2,2-azobisissobutyronitrile (AIBN), and N,N-dimethyl formamide (DMF) were procured from Sigma-Aldrich. Moreover, monobasic potassium phosphate (KH_2_PO_4_) and acetonitrile were purchased from Merck Chemicals. Moreover, quetiapine’s active pharmaceutical ingredient (API) was prepared from commercial sources.

### 2.2 Instrumentation

The Shimadzu Prominence high-performance liquid chromatography (HPLC) system (Shimadzu Corporation, Kyoto, Japan), equipped with an LC-20AD pump, with a DGU-20A degassing agent, a SPD-20A UV–Vis detector, and a CTO-20A column oven, was being applied for all of the below mentioned analyses. Moreover, LabSolutions software version 5.51 has been used for all of the data analysis and subsequent processing. Also, a C18, end-capped (250 × 4.6) mm, 5-µm liquid chromatography column was applied for measuring the concentration of the drug. In addition, a Spectrum 100 FT-IR spectrometer (PerkinElmer Co., Ltd., United States); a CR3i centrifuge (Thermo Fisher Scientific Inc., United States); a CHZ-82 constant temperature water bath oscillator (Fuhua Instrument Co., Ltd., China); an FEI Quanta 200 scanning electron microscope (Thermo Fisher Scientific, Netherlands); and a KQ2200B sonic device with a frequency and temperature controller (Kunshan Ultrasonic Instrument Co., Ltd., China) were used to perform the related processes. Finally, a ZRS-8G dissolution analyzer (Tianda Tianfa Technology Co., Ltd., China) was applied for the examinations.

### 2.3 Preparation of molecular imprinted polymers

Both MIPs and non-imprinted polymers (NIPs) have been synthesized by the precipitation polymerization method, along with UV irradiation (Motaharian et al., 2020; He et al., 2021). For preparation of the MIPs, quetiapine (383 mg, 1 mmol) was used as the template molecule and MAA (793 mg, 4 mmol; as the functional monomer) and EGDMA (3.77 ml, 20 mmol; as cross-linker) were dissolved in 50 ml of DMF in a thick-walled glass tube. The solution was stirred for 4 h to form a complex. Then, AIBN (50 mg, 0.347 mmol) was added to initiate the polymerization process (Motaharian et al., 2020; He et al., 2021). Subsequently, the mixture was sonicated for 5 min. After sonication, the mixture was purged with N_2_ gas for 5 min to remove the dissolved oxygen.

Then, the reaction matrix was stirred in a UV cabinet at 366 nm for 24 h. After drying in an aerobic atmosphere, the prepared MIP particles were refluxed in a Soxhlet extractor apparatus using methanol–acetic acid (9:1 v/v) three times to remove the quetiapine residues from the substrates. The polymers were dried at room temperature for 72 h. The obtained polymer matrix was powdered by grinding with a mortar. Simultaneously, an NIP (as reference) was synthesized and treated under the same conditions without the use of quetiapine.

### 2.4 Characterization of the molecular imprinted polymer composite

#### 2.4.1 Scanning electron microscopy analysis

The morphologies of both MIP and NIP were studied with SEM. The SEM photographs were prepared by an FEI ESEM QUANTA 200 (United States). The surfaces and cross-sections of the MIP and NIP matrix were made conductive by deposition of a gold layer on the samples in a vacuum chamber. Figure 3 shows an SEM image of the polymer matrixes. Moreover, the morphologies of the samples were observed under SEM scanning at a voltage of 25 kV. Also, the *ImageJ 1.53 k* open-source Java image processing program was applied for data extraction from the SEM images.

#### 2.4.2 FT-IR analysis

The FT-IR spectra of both the MIP and NIP (as reference) were prepared by the FT-IR analyzer. To perform it, a certain amount (about 5 mg) of MIP and NIP powders and 100 mg powder of KBr were mixed and ground properly. Then, the mixture was pressed into 1-mm pellets. The obtained samples were measured on a Spectrum 100 FT-IR spectrometer. The FT-IR spectra of the MIPs and the NIPs were plotted by recording from 4,000 to 400 cm^−1^, with a resolution of 2 cm^−1^, by applying a pellet of potassium bromide as the reference.

#### 2.4.3 Drug loading into the molecular imprinted polymer

Quetiapine was loaded into the polymer matrixes by immersing 10 mg MIPs in 10 ml of quetiapine solution (25 μg/ml) and soaked for 30 min. Then, it was placed on a shaking table for 24 h at room temperature. Finally, the mixture was filtered and dried under vacuum at 40°C.

#### 2.4.4 Swelling by molecular imprinted polymer and non-imprinted polymer particles

A total of 10 mg of dry polymer particles were incubated in buffers 1.2, 5, 5.8, and 6.8 until the nanoparticles reached equilibrium. Then, those were centrifuged at 4,000 rpm for 15 min. The abovementioned solution was discarded, and the swollen particles were weighted.

The swelling ratio was calculated by using Eq. 1:

$$Swelling\;ratio\left(\%\right)=\left[\left(Ws\;–\;W_{d}\right)/W_{d}\right]\times 100.$$ (1)

where W_d_ is the weight of the dry polymer and W_s_ is the swollen polymer at a given time.

### 2.5 Data analysis

In order to study the quetiapine transportation mechanism by the MIPs and NIPs, three diffusion models containing the Higuchi model, the first-order pattern, and the zero-order pattern were applied. The kinetic studies were performed by plotting the cumulative values of the drug (in percentage) per time (in hours). The correlation coefficient (r) for each of the kinetic models was calculated to find the model that was followed.

The zero-order model relation (Eq. 2) (concentration per time):

$${Q=Q}_{0}{+K}_{0}t.$$ (2)

The relation of the Higuchi model (Eq. 3) (concentration per square root of time):

$$Q_{t}/Q_{0}{=K}_{H}t^{1/2}.$$ (3)

The relation of the first-order model (Eq. 4) (logarithm of the concentration per time):

$$\mathrm{Log}\;Q_{t}{=logQK}_{t},$$ (4)

where Q_t_ is the amount diffused (mg) per time t (h), Q_0_ is the initial amount in the donor compartment (µg). K_0_ is the zero-order constant (µg h^−1^), K_1_ is the first-order constant (µg h^−1^), and K_H_ is Higuchi’s constant (µg h^1/2^). The correlation coefficient (r) for each kinetic model has been calculated to determine the model that was followed.

#### 2.5.1 Adsorption experiments

For the adsorption experiment, 10.0 mg of the MIPs or NIPs were suspended in 10.0 ml of quetiapine solution (2.5–50 µg/ml) and 2 ml of phosphate buffer at different pHs for a time period between 2 min and 240 min and measured. Then, the sample solutions were filtered by a micro-membrane, and the amount of the drug in the filtrate was determined by HPLC according to the procedure described in the HPLC section. The binding capacity of both NIPs and MIPs and the selectivity factor were calculated by Eqs. 5 and 6, respectively.

$$Qt=\left(C_{0}{-C}_{t}\right)V/m,$$ (5)

$$\alpha =Q_{MIP}/Q_{NIP},$$ (6)

where Q_t_ (μmol g^−1^) is the adsorption capacity at different times, C_0_ (mmol L^−1^) is the initial concentration of quetiapine, Ct (mmol L^−1^) is the concentration of the drug at time t, V (in liters) is the volume of the initial quetiapine drug solution, and m is the mass of the NIP and MIP. Finally, α is the selectivity factor for both NIPs and MIPs, Q_NIP_ (μmol g^−1^) is the adsorption capacity of the NIP, and Q_MIP_ (μmol g^−1^) is the adsorption capacity for MIP.

#### 2.5.2 Release experiments

The most comprehensive equation for describing drug release is the semi-experimental known from the expression of the law of power, developed by Korsmeyer et al. in 1983 (Higuchi, 1963; Korsmeyer et al., 1983). The Korsmeyer–Peppas equation is expressed as follows:

$$\log\left(M_{t}/M_{\infty }\right)=logk+nlogt,$$ (7)

where M_t_ is the amount of the drug released during time t and M_α_ is the released amount at time α. Therefore, the M_t_/M_α_ is the fraction of the drug released at time t. Also, K is the kinetic constant and n is the diffusion exponent. Due to these factors, the plot between the logarithms of M_t_/M_α_ per the logarithm of time would be linear if the release follows the Korsmeyer–Peppas equation. Therefore, the slope of this plot represents the “n” value. The relations of the other models for release experiments are the same as for adsorption models.

### 2.6 High-performance liquid chromatography method

The chromatographic determination of quetiapine concentration and its release was carried out by a Shimadzu HPLC system equipped with a UV/VIS detector set to 230 nm. The separation was performed by a C18 (4.6 mm, 250 mm, 5 μm) liquid chromatography column. The injection volume was 50 μl, the flow rate was 1.3 ml min-^1^, and the column temperature was fixed at 25°C by using the column oven. An isocratic method was used for the elution, and the mobile phase was phosphate buffer (pH = 5.6)/ACN/methanol (39: 7: 54), v/v percentage, where the buffer was prepared by dissolving 10 mM of monobasic potassium phosphate in 1 L of purified water set at a pH of 5.6 by phosphoric acid.

### 2.7 Quantum chemical calculations

The separated forms of the quetiapine drug and the polymer of MAA-EGDMA were designed as input files and were then optimized to yield the most stable energy minima. Then, the complex systems of the polymer in each form with the drug were subsequently designed and put under related calculations. The Gaussian 03 quantum chemical package was applied to perform the required calculations (Korsmeyer et al., 1983). Also, the related parameters were reached out accordingly. Moreover, investigations on all stationary points in addition to the other required calculations were carried out by using the B3LYP/6-31g(d) level of theory (Higuchi, 1963; Frisch, 2004), which were confirmed to be suitable for such investigations (Barhoumi et al., 2015; Kumsampao et al., 2020). The global electron density transfer (*GEDT*) has been calculated by applying the following relation (Siadati and Nami, 2016; Parlak et al., 2019):

$$GEDT=\sum q_{A},$$ (8)

where q_A_ is the net Mulliken charge and the sum of the entire atoms of the gaseous species.

Also, the energy of adsorption E_ad_ was calculated by using the following relation (Kącka-Zych and Jasiński, 2020; Mohtat et al., 2018; Kula et al., 2021):

$$E_{ad}{=E}_{sys}–\left(E_{Sorbent}+E_{TM}\right),$$ (9)

where E_sys_, E_Sorbent_, and E_TM_ are the energy of the system, the energy of the isolated sorbent, and the energy of the template molecule, respectively.

## 3 Results and discussion

### 3.1 Structural characterization of the MIP-BC

#### 3.1.1 FT-IR spectroscopic analysis

**FIGURE 2:**
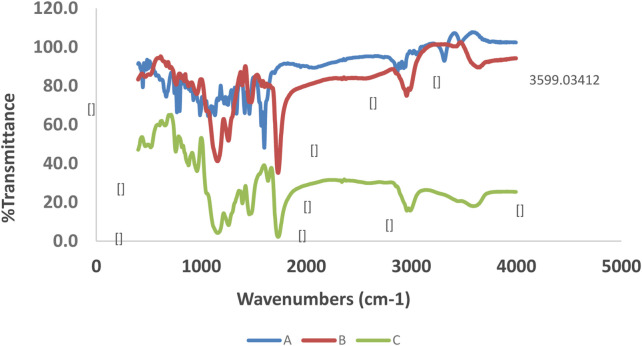
FT-IR spectra of quetiapine [**(A)**; blue line], drug-loaded MIP [**(B)**; brown line], and unloaded MIP [**(C)**; green line].

As shown in Figure 2, the C=O and O-H stretching and bending vibrations at 1,732 cm^−1^, 3,599 cm^−1^, and 1,163 cm^−1^ in the loaded MIPs were shifted to 1,735 cm^−1^, 3,603 cm^−1^, and 1,166 cm^−1^ in the unloaded MIPs, respectively. Moreover, in the unloaded MIPs, a vibration was observed belonging to a new band with low intensity at 2,995 cm^−1^, which was also presented at 2,958 cm^−1^ in the same unloaded MIP. Moreover, some other absorption peaks corresponding to the MIPs were detected as follows: 1,144 cm^−1^, 1,249 cm^−1^ (symmetric and asymmetric ester C–O stretch bands), 1,636 cm^−1^ (stretching vibration of residual vinylic C–C bonds), and 985 cm^−1^ (out-of-plane bending vibration of vinylic C–H bond).

#### 3.1.2 Morphological structure analysis

**FIGURE 3:**
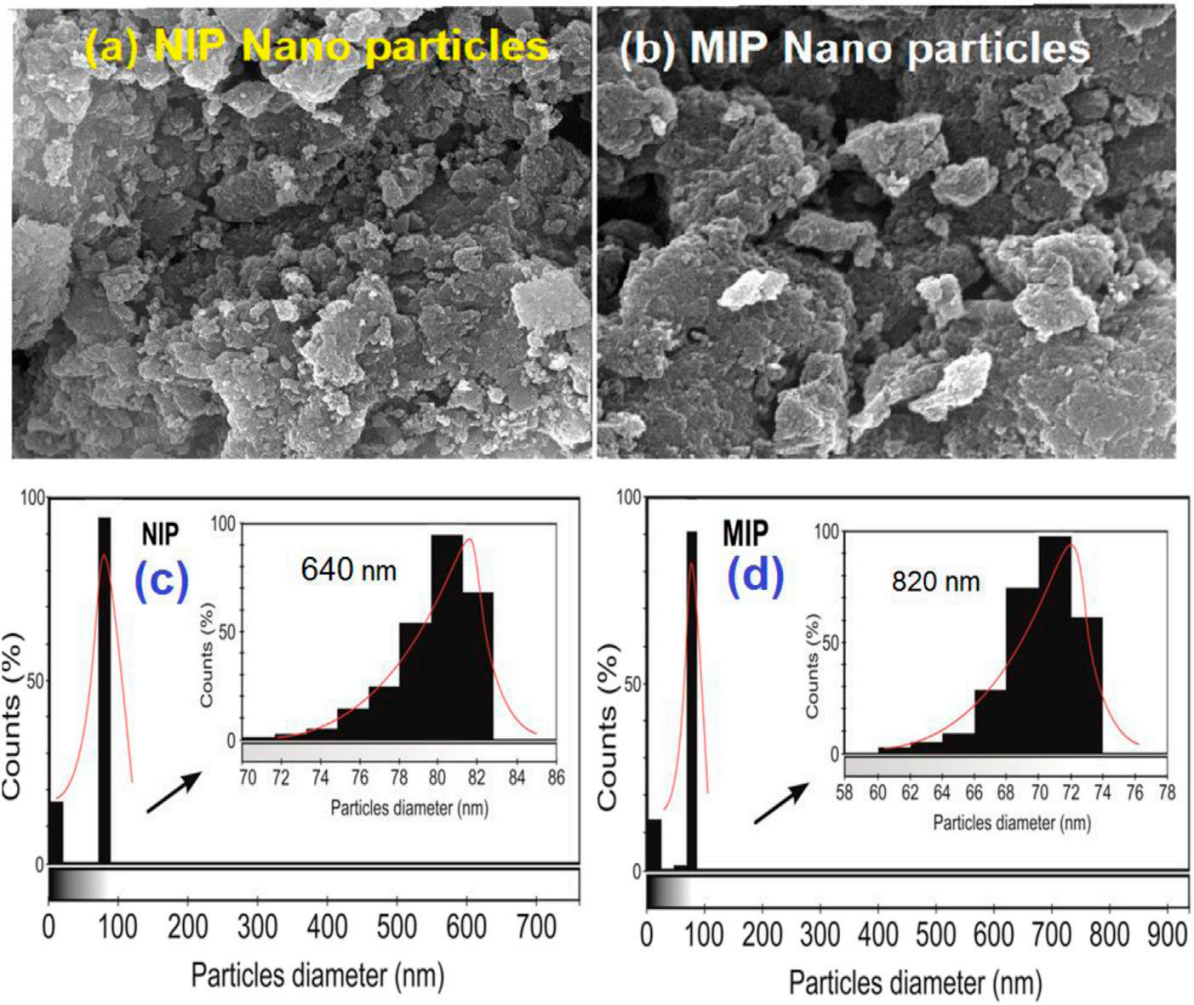
SEM images of **(A)** NIP nanoparticles, **(B)** MIP nanoparticles, and the particle diameter count diagrams prepared by ImageJ 1.53 k software for **(C)** NIP and **(D)** MIP.

As shown in Figure 3, the surfaces of the MIP and NIP particles are monitored by using SEM photographs. These figures show that both MIP and NIP particles have uniform and almost spherical morphologies. Moreover, the histograms obtained by the ImageJ 1.53 k program confirmed that the average particle sizes are about 81 nm and 70 nm for NIP and MIP, respectively. Indeed, the SEM images represented here show the uniform and regular texture of the particles. These also confirm that both MIPs and NIPs are nano-sized due to their nano-scale diameters. Also, no considerable difference in terms of the surface morphology between the NIP and MIP was observed. Thus, loading the quetiapine of the polymer or using this drug as the molecular template in the synthesis of MIP has not resulted in a considerable change in its surface morphology. Moreover, oligomerization of particles has been observed in some parts of the picture. Such an occurrence might be due to the increase in surface reactivity and the tendency for oligomerization in the nano-scale processing.

#### 3.1.3 Measurement of swelling of molecular imprinted polymer and non-imprinted polymer particles

The percentage of swelling of MIP and NIP polymers is given in Table 1. The results show that the rate of swelling of MIP is higher than that of NIP due to the presence of regular and numerous sites in the MIP. On the other hand, an increase in polymer inflammation with increasing pH could be due to ionization of carboxylic groups of the acrylate structure and electrical repulsion between negative charges. It subsequently causes the polymer to become more hydrophilic and allows the water molecules to penetrate the polymer structure.

**TABLE 1:** Swelling amounts of MIPs and NIPs by solvents.

Polymer	W_1_ (g)	W_2_ (g)	%swelling	pH
MIP	0.01	0.041	75	6.8
NIP	0.01	0.020	51	6.8
MIP	0.01	0.024	58	5.8
NIP	0.01	0.018	44	5.8
MIP	0.01	0.017	43	5
NIP	0.01	0.015	35	5
MIP	0.01	0.014	28	1.2
NIP	0.01	0.011	13	1.2

### 3.2 Batch adsorption experiments

#### 3.2.1 Effect of pH on drug absorption

**FIGURE 4:**
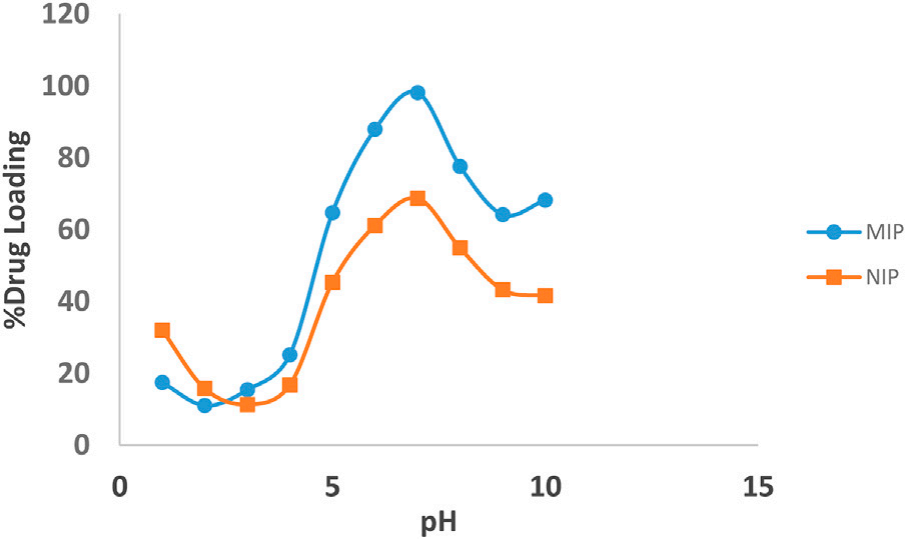
Effect of pH on the loading percentages of the drug on the MIP (blue line), and NIP (brown line).

The binding interactions between quetiapine and MIP are attributed both to the hydrogen bonds and the electrostatic forces. The effect of pH on the absorption of quetiapine to the substrates was examined by varying the pH of the solution from 2 to 10. Also, several batch experiments were performed by loading 10 mg of the MIP and NIP in quetiapine solutions (10 ml, 50 μg ml^−1^) under particular levels of pHs. The results indicated that the pH plays an important role in the drug-loading capacity (Figure 4).

The highest amount of rebinding on the MIP was observed at pH = 7. Moreover, the percentage of quetiapine loading has increased with increasing the pH from 2 to 7, while, with a further increase of pH from 7 to 10, it decreased. Also, at pH = 7, the loading amount of the MIP was about 30% more than that of the NIP. Weaker effects were observed at lower or higher pH values, which might be related to the protonation of the functional groups of quetiapine and deprotonation of carboxyl groups of the polymer, respectively.

#### 3.2.2 Adsorption kinetic studies

The kinetics of the adsorption of quetiapine by the MIP and NIP were investigated *via* pseudo-first-order and pseudo-second-order models. To perform this, the dried polymer (10 mg) and quetiapine solution (10 ml, 50 μg/ml, pH = 7) were placed in different centrifuge tubes, and each tube was shaken for 2–20 min. By increasing the exposure time, adsorption efficiency slowly increased and remained constant within 5 min. The rate constant of adsorption is determined by the first-order rate expression:

$$\log\left(q_{e}-q_{t}\right)=\log q_{e}-\frac{k_{1\;}}{2.303t},$$ (10)

where $q_{e}$ and $q_{t}$ are the amounts of quetiapine (mg g^−1^) adsorbed at the equilibrium and at time t (min), respectively. Also, k_1_ is the rate constant of adsorption (min^−1^). The values of k_1_ for quetiapine adsorption on the MIP were defined by the plot of $\log\left(q_{e}-q_{t}\right)$ against t. The values of k_1_ and $q_{e}$ are presented in Table 2.

**TABLE 2:** Kinetic parameters for pseudo-first-order equation and pseudo-second-order equation at 25°C.

	q_e,cal_ (mg/g)	K_1_ (min^−1^)	K_2_ (g/(mg min))	R^2^
Pseudo-first-order NIP	1.421	0.1011	--	0.5264
Pseudo-first-order MIP	3.467	0.0877	--	0.8584
Pseudo-second-order NIP	22.4215	--	0.1069	0.9958
Pseudo-second-order MIP	68.9655	--	0.0134	0.9937

Also, the second-order kinetic model is expressed as follows:

$$\frac{t}{q_{t}}=\frac{1\;}{q_{e}^{2\;}\times k_{2}}+\frac{t}{q_{e}}.$$ (11)

**FIGURE 5:**
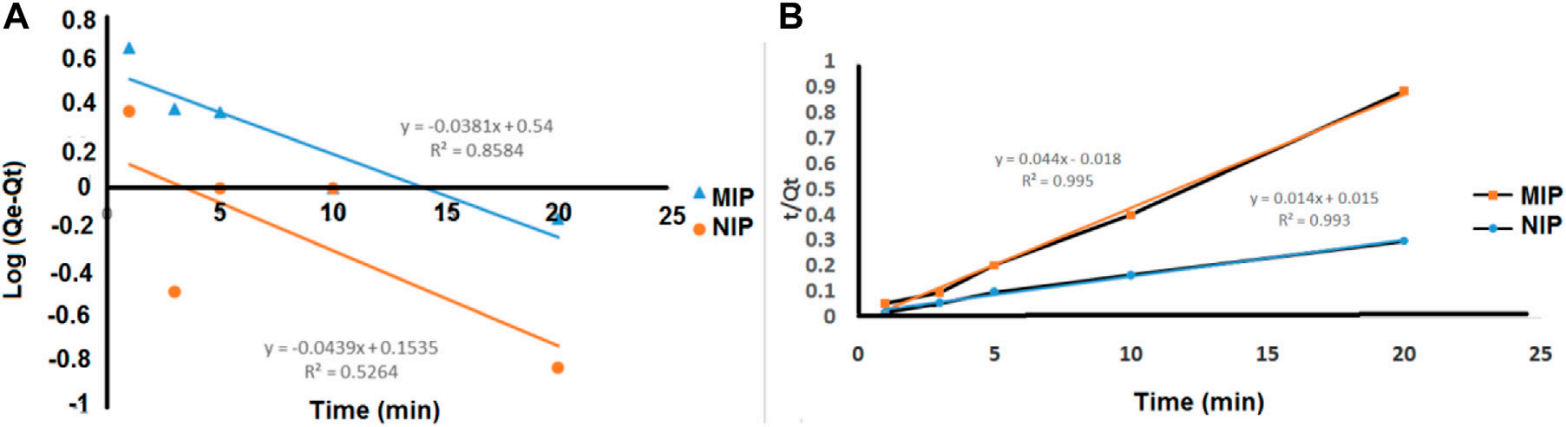
**(A)** Pseudo-first-order and **(B)** pseudo-second-order kinetic models for the adsorption of quetiapine on the MIP and NIP.

The second-order constant k_2_ (g mg^−1^ min^−1^) and the equilibrium adsorption capacity q_e_ could be determined experimentally by the slope and intercept of the plot t/q versus t (Figure 5). In the second-order kinetic model, the calculated correlations were closer to unity. Therefore, the adsorption kinetics is more fitted to the second-order kinetic model.

### 3.3 Release experiments

**FIGURE 6:**
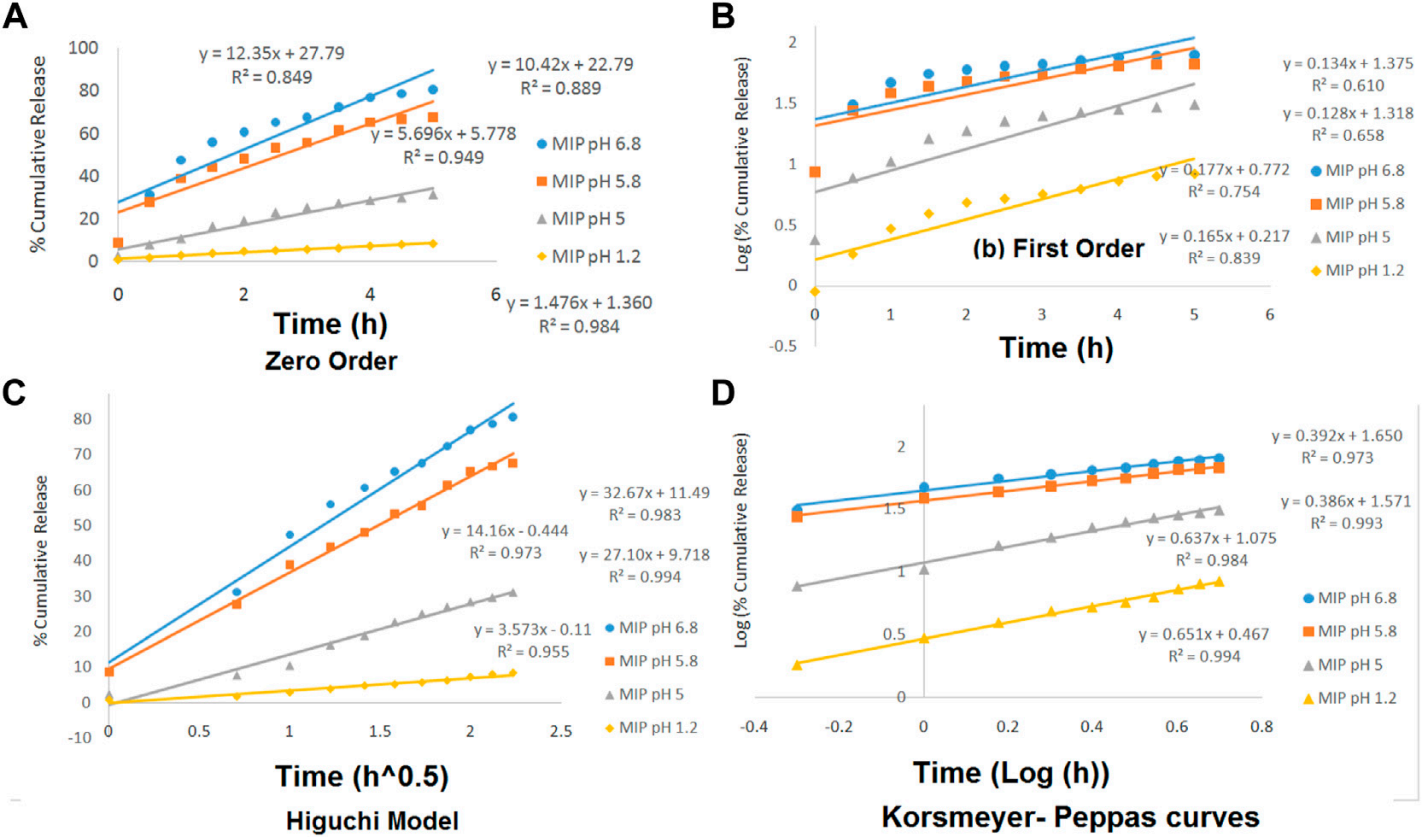
Zero-order **(A)**, the first-order **(B)**, the Higuchi model **(C)**, and the Korsmeyer–Peppas **(D)** curves for MIP at various pH levels of 1.2, 5, 5.8, and 6.8.

The release kinetics of the drug was studied based on four models of zero order, first order, Higuchi, and Korsmeyer–Peppas. The data obtained from those models were evaluated based on the correlation coefficient (R^2^). The mechanism of drug release from the MIP has been described in the theoretical section. However, in this section, the drug release kinetics has been investigated by plotting different kinetic models containing the zero-order, the first-order, the Higuchi, and the Korsmeyer–Peppas models in various pH values (Figure 6).

The linearity of the curves in Figure 6 and also the numerical data presented in Table 3 indicate that the kinetics of the drug release from the MIP follows the Higuchi model at the pHs of 5.8 and 6.8 and the Korsmeyer–Peppas model at the pHs of 1.2 and 5.

**TABLE 3:** Release kinetic studies of the release of the drug from the MIP.

pH	Zero-order		First-order		Higuchi		Korsmeyer–Peppas	
	K	R^2^	K	R^2^	K	R^2^	N	R^2^
1.2	1.477	0.9842	−0.3809	0.8399	3.5739	0.955	0.6517	0.9945
5	5.6961	0.9499	−0.4092	0.7541	14.168	0.9731	0.6371	0.9842
5.8	10.429	0.8891	−0.2950	0.6586	27.103	0.9943	0.3865	0.9935
6.8	12.35	0.8493	−0.3095	0.6105	32.676	0.983	0.3929	0.9739

Furthermore, the values of n in the Korsmeyer–Peppas equation were 0.6517 (in pH = 1.2) and 0.6371 (in pH = 5). It indicated that the release kinetics has followed the non-Fickian diffusion, which might be due to the combination of swelling and erosion of the polymeric matrix.

### 3.4 Theoretical calculations and the binding energies

As given earlier, the quantum chemical calculations are applied to give a better view of the interactions between the host (polymer matrix) and the guest (quetiapine as the drug). With the aid of such investigations, we could study the molecular and atomic behaviors of sub-molecular fragments and other chemical species. To do that, in the first step, we have designed each of the separated species containing the quetiapine drug and the complex molecular segment as the polymer matrix. Then, the designed geometrical structures were created as input files and subjected to further quantum chemical calculations to yield any stable or meta-stable species as output files. Subsequently, the optimized structure of quetiapine was placed near the polymer segment to yield the host–guest complex systems. Then, the energy saddles of the best geometrical places were found, and the theoretical level of calculation was evaluated to the B3LYP/6-31G(d). Finally, the structural parameters, sub-molecular and atomic orientations, Mulliken charges, PES, and FMO results were extracted, accordingly. The results of the theoretical calculations were shown to have good agreement with experimental ones, revealing strong interactions between the polymer matrix and the drug.

**FIGURE 7:**
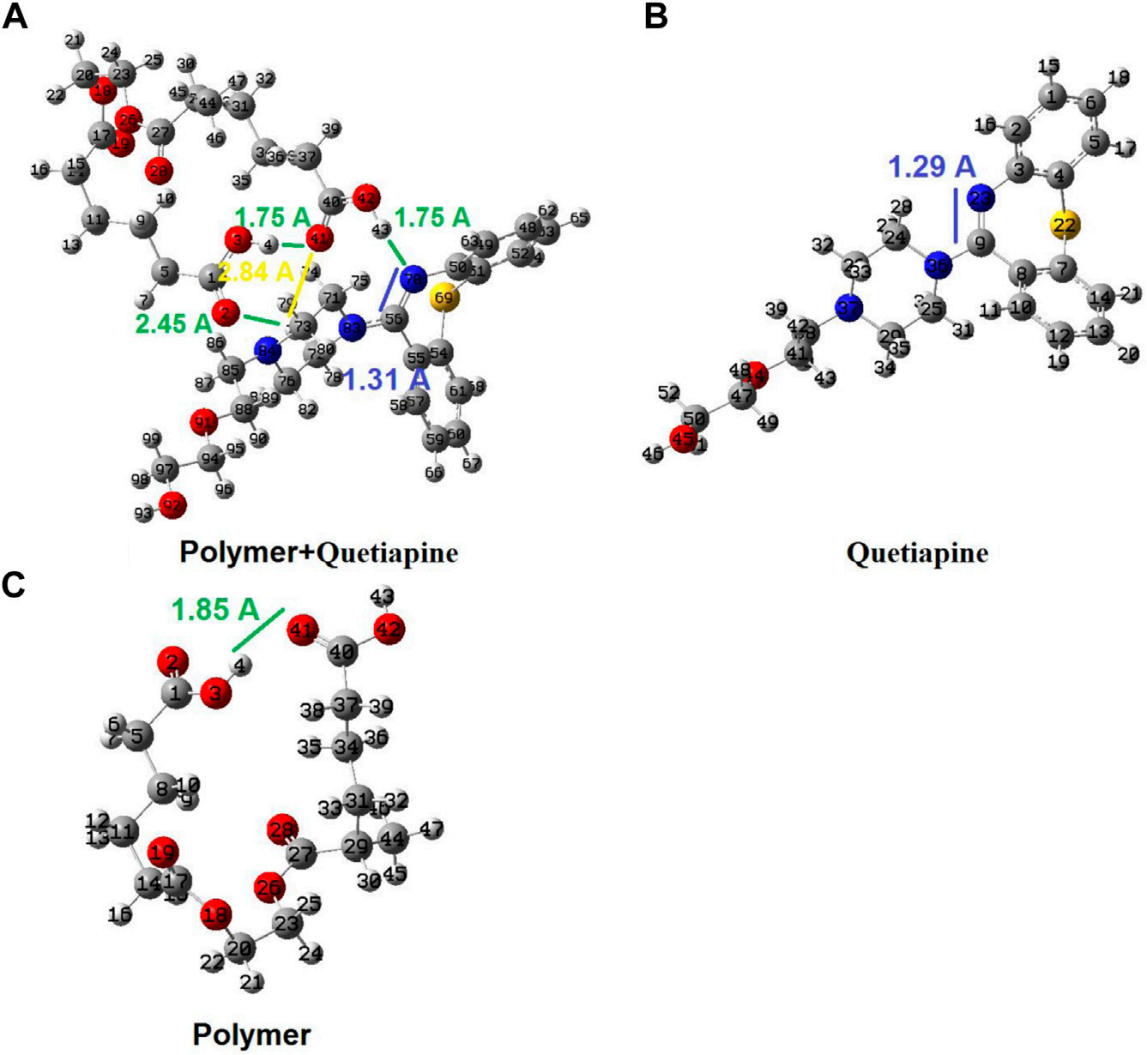
Geometrical structures for the isolated polymer, isolated quetiapine (drug), and the complex of the polymer–quetiapine system optimized by quantum chemical calculations.

As shown in Figure 7, there is only one hydrogen bond (H4-O41 with 1.85 Ǻ) in the theoretically simulated free-polymer fragment (Figure 7C), while there are several hydrogen bonds and interactions between the conjugated polymer–quetiapine system (such as H4-O41 = 1.75 Ǻ; H43-N70 = 1.75 Ǻ; H77-O2 = 2.45 Ǻ; H77-O41 = 2.84 Ǻ). The considerably higher number of hydrogen bonds between the polymer (host fragment) and quetiapine drug (guest part) confirms a strong interaction between the polymer matrix and quetiapine drug. Moreover, the C56 = N70 bond length increases from 1.29 Ǻ in the isolated quetiapine to 1.31 Ǻ in the host–guest complex, which shows that this bond is being weakened to transfer the electron density to the H43 of the host. Also, the H4–O41 hydrogen bond length decreases from 1.85 Ǻ in the isolated polymer to 1.75 Ǻ in the host–guest complex, indicating more compression of the polymer in the complex, compared to its isolated form.

**FIGURE 8:**
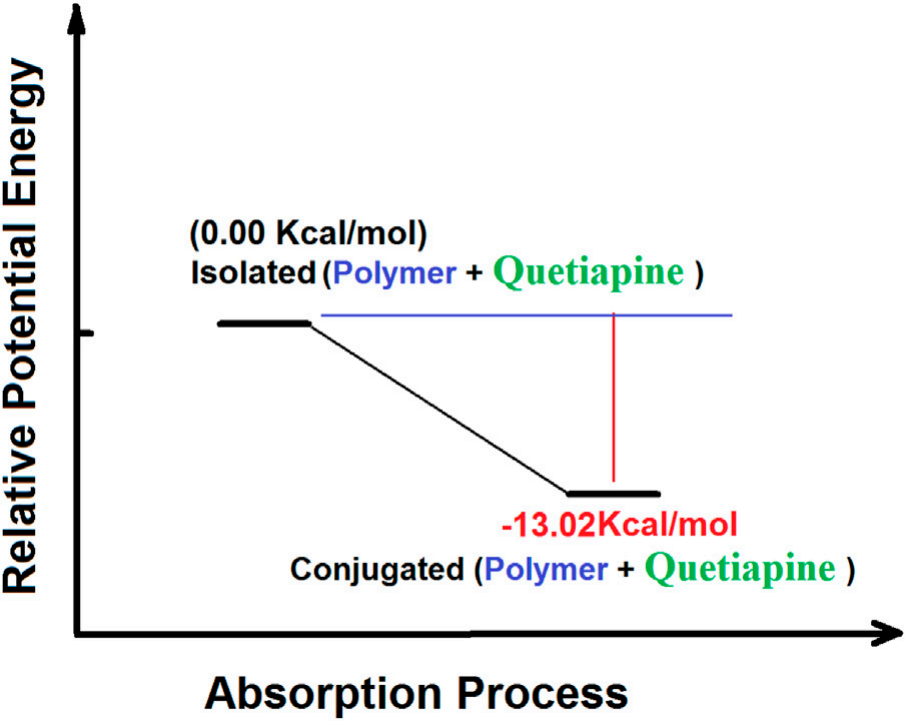
Potential energy surface relating to the absorption processes of quetiapine by the polymer matrix calculated by the B3LYP quantum chemical method.

On the other hand, the results of the PES calculations (Figure 8) show that the absorption of quetiapine by the polymer matrix as the host releases an amount of −13.02 kcal mol^−1^. Thus, the PES studies indicate that the absorption process of quetiapine by the polymer is favorable in terms of thermodynamics. Such results would confirm the outcome of the theoretical calculations of the structural analysis in fast adsorption of quetiapine by the polymer.

**FIGURE 9:**
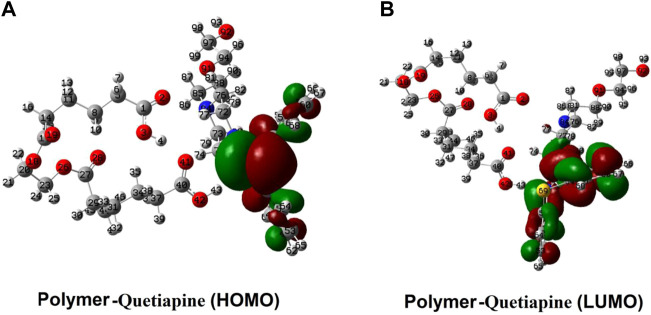
Spatial distribution of the FMOs of quetiapine (the guest) and the polymer matrix (the host).

As presented in Figure 9, the distribution of the frontier molecular orbitals (FMOs) indicates that the HOMO and LUMO orbitals are mainly positioned on the rings of the quetiapine (guest) side. Somehow, there is no observable sign of FMOs in the host, while in the case of the guest (quetiapine), major parts of FMOs could be found in the aromatic rings and the central seven-membered ring. On the one hand, it shows that there are no significant electron transfers or covalent bond formations between the host and the drug. On the other hand, such FMO arrangements reveal that even under the existing state conditions such as *hv* irradiations (LUMO system), no considerable change happens in view of the type of interactions between the host and the guest. Moreover, the *GEDT* indices for the polymer in the adsorption process are about −0.091, which indicates that a small amount of electron charge is transferred from the drug into the polymer.

## 4 Conclusion

The quetiapine MIPs were prepared by the precipitation polymerization approach. The morphology of the polymer was investigated by SEM, indicating that the MIP and NIP particles are at the nanoscale. The results of quetiapine loading studies show that the MIP nanoparticles have a higher quetiapine loading capacity than NIPs (more than 30% at pH = 7). Moreover, the adsorption experiments show that the highest amount of rebinding on the MIP is at pH = 7. Somehow, the percentage of quetiapine loading increases with increasing the pH from 2 to 7, while, with a further increase of pH from 7 to 10, it decreases. Also, at pH = 7, the loading amount of the MIP was about 30% more than that of NIP. Weaker effects, which are observed at lower or higher pH values, might be related to the protonation of the functional group of quetiapine and deprotonation of carboxyl groups of the polymer, respectively. Also, those investigations indicate that the kinetics of adsorption both for the cases of MIPs (R^2^ = 0.9937) and NIPs (R^2^ = 0.9958) are fitted with the pseudo-second-order model. In addition, the kinetics of the drug release from MIP follows the Higuchi model at the pHs of 5.8–6.8 and the Korsmeyer–Peppas model at the pHs of 1.2–5.

Our results showed that MIPs could be favorable devices for drug delivery systems, at least in the case of quetiapine.

Finally, in order to investigate the adsorption behavior of quetiapine on the surface of the polymer matrix, we have used theoretical quantum chemical methods. To do so, the polymer–drug (host–guest) system was studied by the DFT approach. The results of the geometrical optimizations as well as the binding energies showed the presence of some hydrogen bonds and polar interactions between the polymer system. Also, the PES data indicated that the formation of the polymer–quetiapine complex releases a −13.02 kcal mol^−1^ of energy, showing a spontaneous process in terms of thermodynamics.

## Data Availability

The original contributions presented in the study are included in the article/Supplementary Material; further inquiries can be directed to the corresponding authors.
